# Mining functional gene modules by multi-view NMF of phenome-genome association

**DOI:** 10.1186/s12864-024-11120-5

**Published:** 2025-01-09

**Authors:** Xu Jin, WenQian He, MingMing Liu, Lin Wang, YaoGong Zhang, YingJie Xu, Ling Ma, YaLou Huang, MaoQiang Xie

**Affiliations:** 1 College of Software, Nankai University, TianJin, China; 2 TianJin International Joint Academy of Biomedicine, TianJin, China

**Keywords:** Nonnegative matrix factorization, Gene module mining, Phenotype ontology, Hierarchical structure

## Abstract

**Background:**

Mining functional gene modules from genomic data is an important step to detect gene members of pathways or other relations such as protein-protein interactions. This work explores the plausibility of detecting functional gene modules by factorizing gene-phenotype association matrix from the phenotype ontology data rather than the conventionally used gene expression data. Recently, the hierarchical structure of phenotype ontologies has not been sufficiently utilized in gene clustering while functionally related genes are consistently associated with phenotypes on the same path in phenotype ontologies.

**Results:**

This work demonstrates a hierarchical Nonnegative Matrix Factorization (NMF) framework, called Consistent Multi-view Nonnegative Matrix Factorization (CMNMF), which factorizes genome-phenome association matrix at consecutive levels of the hierarchical structure in phenotype ontology to mine functional gene modules. CMNMF constrains the gene clusters from the association matrices at two consecutive levels to be consistent since the genes are annotated with both the child-level phenotypes and the parent-level phenotypes in two levels. CMNMF also restricts the identified gene clusters to be densely connected in the phenotype ontology hierarchy. In the experiments on mining functionally related genes from mouse phenotype ontology and human phenotype ontology, CMNMF effectively improves clustering performance over the baseline methods. Gene ontology enrichment analysis is also conducted to verify its practical effectiveness to reveal meaningful gene modules.

**Conclusions:**

Utilizing the information in the hierarchical structure of phenotype ontology, CMNMF can identify functional gene modules with more biological significance than conventional methods. CMNMF can also be a better tool for predicting members of gene pathways and protein-protein interactions.

**Supplementary Information:**

The online version contains supplementary material available at 10.1186/s12864-024-11120-5.

## Background

Functional gene modules are often identified from genomic data to find genes with same functions, involved in same pathway or interacting with each other. Traditional methods mine functionally related genes via classical clustering algorithms. K-means and AHC (Agglomerative Hierarchical Clustering) are the most frequently used clustering algorithms to cluster gene expression data. Recently, NMF and its variants have also been successfully adopted for clustering gene expression [1] and gene-phenotype association data [2], owing to its inherently good interpretability and good performance.

More recently, multi-view NMF-based methods are proposed. Singh proposed collective NMF (ColNMF), which can get more consistent gene clustering result by using a shared coefficient matrix but different basis matrices across different views [3]. Zhang and Zhou proposed a multiple NMF framework to integrate multiple types of genomic data to jointly identify microRNA-gene regulatory modules [4]. Additionally, NMF-based methods integrated with some structural information were also proposed and achieved better performance. Pehkonen adopted NMF to analyze association data between gene and Gene Ontology (GO), in which the association matrix is enriched according to the “true path rule” [5] of gene ontology hierarchy [6]. Hwang and Kuang proposed a nonnegative matrix tri-factorization method to cluster phenotypes and genes simultaneously [2]. HPMF focuses on predicting missing traits for plants which incorporates hierarchical phylogenetic information into matrix factorization [7]. Multi-view NMF can explore the consistent and complementary information hidden in different views, thus leading to better gene clustering performance.

In the context of gene clustering, one effective structural feature that has not been integrated with multi-view NMF is the hierarchical structure of Human Phenotype Ontology (HPO) [8], which is potentially helpful for clustering genes. Some NMF-based methods utilize the hierarchical structure in HPO as graph constraint. Graph Regularized Nonnegative Matrix Factorization (GNMF) [9] incorporates the Laplacian constraint of the phenotype graph into NMF to enforce the correlated phenotypes to share similar latent representations, thus possessing similar prioritized gene lists. Moreover, by introducing weighted graph constraint varying with the depth of phenotypes in HPO, GC$^2$NMF [10] attaches more importance to lower-level phenotypes whose associations are more informative.

**Fig. 1:**
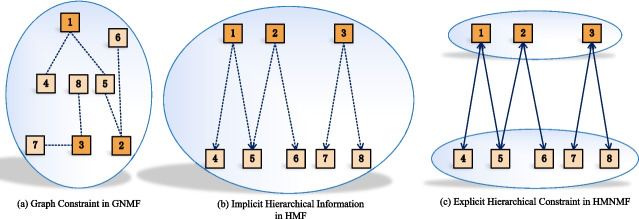
Illustration of Graph Constraint, Implicit Hierarchical Information and Hierarchical Constraint. **a** Graph constraint is a global similarity constraint in HPO hierarchy. **b** Implicit hierarchical relation can be used as a unidirectional regularization between parent-level and child-level, in which child-level embedding can be calculated by embeddings of its parent and itself. **c** Hierarchical constraint in CMNMF is a bidirectional regularization between two levels of HPO hierarchy, in which two phenotype embeddings will be similar if there is a parent-child relationship in HPO

Despite the effectiveness of graph constraint, it is incapable of fully depicting the hierarchical structure in HPO. As shown in Fig. 1a, graph constraint is a global similarity constraint, which views HPO as a flat and uniformly distributed network where two levels of phenotypes are located in common latent space thus indivisible from each other. Specifically, the latent vectors of two levels of phenotypes are calculated simultaneously by factorizing the global correlation matrix, therefore, the complementary information of two levels of phenotypes can not be fully exploited.

Most methods aim to integrate implicit hierarchical information rather than hierarchical constraint into NMF. A classical one is Hierarchical Matrix Factorization (HMF) [11], which incorporates the hierarchical information of phenotypes into matrix factorization, where the embedding of each child-level phenotype is calculated by summing the embeddings of itself and its parent-level phenotypes. Besides, a novel collaborative filtering method with hierarchical side-information (HSCF) [12] even integrates three different strategies of utilizing hierarchical structure together, which can better exploit the complementary information among adjacent levels but also unable to fully explore the pair-wise hierarchical relations in each pair of hierarchically related nodes. The implicit hierarchical information in these methods can explore the latent correlations among hierarchy in some way. However, similar to graph constraint, the complementary information of two levels of gene-phenotype association networks can not be fully explored. Especially for HMF demonstrated in Fig. 1b, the dashed unidirectional arrows indicate the hierarchical information transmitted from parent-level phenotypes to child-level phenotypes. However, the hierarchical information from the reverse direction is omitted since it only considers the incorporation of the latent vectors of parent-level phenotypes.

Some more advanced methods learn underlying hierarchical relations from the networks. Random Partition Matrix Factorization (RPMF) [13] recursively partitions the primitive network into multi-level sub-networks by Random Decision Tree (RDT), where the learned levels of sub-networks can describe the underlying hierarchical relations among nodes. Furthermore, Hidden Hierarchical Matrix Factorization (HHMF) [14] iteratively groups the genes and phenotypes into smaller groups by assuming the latent embeddings of genes and phenotypes conform to consistent distributions. These methods can capture hidden hierarchical relations with the known associations among nodes even though the hierarchical relations are unavailable but still suffers from the inability of fully exploiting the hierarchical structure.

There are recently rare methods incorporating hierarchical constraint into NMF. Hierarchical Probabilistic Matrix Factorization (HPMF), proposed by [15] for link prediction between plants and traits, imposes pair-wise similarity constraint on the latent vectors of each pair of hierarchically related plants, which can take full advantage of the hierarchical structure.

**Fig. 2:**
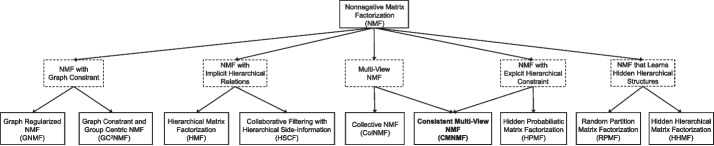
Five variants of NMF and their classical methods. The proposed CMNMF is a hybrid method of both multi-view NMF and NMF with explicit hierarchical constraint

All these NMF variations are summarized to show their relationships in Fig. 2.

In this work, it is assumed that the clusters of genes with different representations in multiple views should be consistent, i.e. genes will be consistently clustered by the associations with phenotypes at different levels (or granularity) of the hierarchy in a phenotype ontology, where each level of the a phenotype ontology provides a different view for gene clustering.

Based on this motivation, the paper presents a multi-view NMF approach called CMNMF (consistent multiple non-negative matrix factorization) for mining functional gene modules, in which the hierarchical structure of phenotype ontologies is introduced as prior knowledge. In detail, a consistency constraint on gene clusters and a hierarchical mapping constraint between phenotypes in two consecutive levels of ontologies are introduced in the loss function. In experiments, CMNMF is applied on gene-phenotype association data of both mouse and human. CMNMF is compared with the baseline methods by measuring the performance of predicting KEGG pathways and protein-protein interactions. Furthermore, GO enrichment analysis by DAVID tool [16] is performed to evaluate the biological significance of the identified gene clusters.

## Methods

### Data preparation

The gene-phenotype associations of mouse were downloaded from Mouse Genome Informatics (MGI) [17], consisting of 15,524 associations between 5,971 phenotypes and 1,350 genes. Furthermore, 3,414 phenotypes at level 7 and their 2,557 parent-level phenotypes at level 6 of phenotype ontology were applied in the experiments followed by the description in [18]. Two versions of mouse protein-protein interaction (PPI) networks were obtained from the BIOGRID (Feb. 2016 and Nov. 2020) [19] and 292 mouse KEGG pathways [20] were applied for the evaluation.

The gene-phenotype associations of human were downloaded from HPO project [21], containing 53,929 associations between 3,280 genes and 5,948 phenotypes. Furthermore, 3,707 phenotypes at level 8 and their parent-level 2,241 phenotypes at level 7 were applied in the experiments. Similar to the mouse data, two versions of human PPI networks were downloaded from the BIOGRID (Feb. 2016 and Nov. 2020) and 296 human KEGG pathways were used for the evaluation.

### Notations

The notations to define the models are summarized in Table 1. Let *n* be the number of genes and *m* be the number of phenotypes, the gene-phenotype associations are represented by a binary matrix $\varvec{A}^{(n \times m)}$ with 1 for entries of known associations and 0 otherwise. $\varvec{G}^{(n \times k)}$ and $\varvec{P}^{(k \times m)}$ represent the cluster indicator matrix of genes and phenotypes respectively, where *k* is the number of clusters. $\varvec{A}_{1}^{(n \times m_{1})}$ and $\varvec{A}_{2}^{(n \times m_{2})}$ denote the association matrix between genes and parent-level/child-level phenotypes respectively, where $m_1$ and $m_2$ are the number of parent-level and child-level phenotypes respectively. The hierarchical structure of phenotype ontology is represented by $\varvec{M}^{(n_1\times n_2)}$.

**Table 1:** Summary of Notations

Notations	Explanations
$\varvec{A}$	Genome-phenome association matrix
$\varvec{A}_1$	Genome-phenome association matrix with phenotype ontology at parent level
$\varvec{A}_2$	Genome-phenome association matrix with phenotype ontology at child level
$\varvec{G}$	Cluster indicator matrix of genes
$\varvec{P}$	Cluster indicator matrix of phenotypes
$\varvec{P}_1$	Cluster indicator matrix of parent-level phenotypes
$\varvec{P}_2$	Cluster indicator matrix of child-level phenotypes
$\varvec{M}$	Hierarchical structure of phenotype ontologies
*n*	The number of genes
*m*	The number of phenotypes
*k*	The number of latent clusters
$m_1$	The number of parent-level phenotypes
$m_2$	The number of child-level phenotypes

### Problem formulation

The objective of factorizing matrix $\varvec{A}$ is to derive the cluster indicator matrix $\varvec{G}$ of genes and the cluster indicator matrix $\varvec{P}$ of phenotypes based on the gene-phenotype associations in $\varvec{A}$. The loss function of factorizing the non-negative matrix $\varvec{A}$ can be defined as:

1 $$\begin{aligned} L_{NMF}(\varvec{G},\varvec{P}) = ||\varvec{A}-\varvec{GP}||^{2}_{F}. \end{aligned}$$

**Fig. 3:**
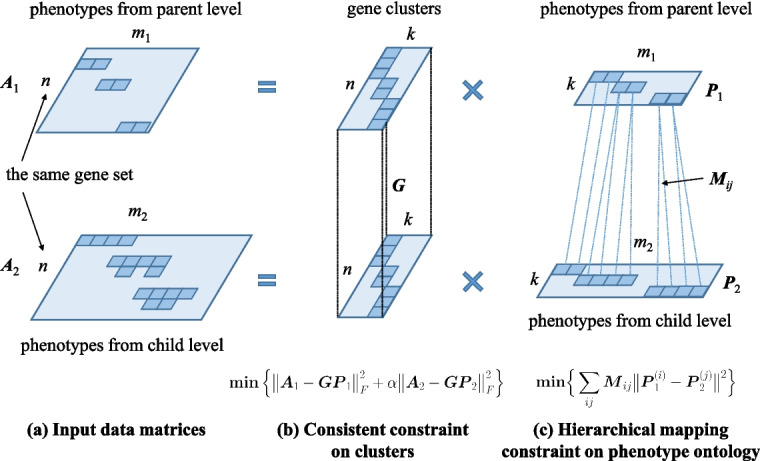
Illustration of the CMNMF framework. **a** The gene-phenotype association matrix are divided into two matrices by the levels of phenotype ontology, and the two matrices share the same factorized cluster indicator matrix of genes. **b** Consistent constraint on factorized cluster indicator matrix of genes. **c** Hierarchical mapping constraint on the phenotypes at parent and child levels

However, the above objective function does not consider the hierarchical structure of phenotype ontology. To address the problem, CMNMF factorizes gene-phenotype association matrices at different levels in the hierarchical structure separately (Fig. 3a). Furthermore, the gene clusters derived from parent-level and child-level phenotypes should be identical (Fig. 3b). In addition, a phenotype mapping constraint is also considered to enforce the learned phenotype embeddings at parent-level and child-level to be consistent with the hierarchical structure (Fig. 3c).

### Loss functions for penalizing inconsistency

Motivated by the assumption mentioned above, the phenotypes are divided into two sets according to their two-level hierarchical structure, then the two gene-phenotype association matrices $\varvec{A}_1^{n\times m_1}$ and $\varvec{A}_{2}^{n\times m_2}$ are set up based on the original gene-phenotype association matrix $\varvec{A}$ and the two sets of phenotypes. The gene cluster indicator matrix $\varvec{G}^{n\times k}$, derived by simultaneous factorization on $\varvec{A}_{1}$ and $\varvec{A}_{2}$, should be consistent, since the genes annotated by phenotypes at adjacent levels should share identical clustering results. $\varvec{G}^{n\times k}$ can be derived by optimizing the following loss function:

2 $$\begin{aligned} {L_C}(\varvec{G},\varvec{P}_1,\varvec{P}_2) = \left\| \varvec{A}_1 - \varvec{G{P}}_1 \right\| _F^2 + \alpha \left\| {\varvec{A}_2} - \varvec{GP}_2 \right\| _F^2 , \end{aligned}$$

where $\alpha$ is a hyper-parameter to balance the two terms of matrix factorization. To utilize the hierarchical mapping relationships between phenotypes at parent-level and child-level, the clustering results of hierarchically related phenotypes are enforced to be similar as follows,

3 $$\begin{aligned} {L_H}(\varvec{P}_1,\varvec{P}_2) = \sum \limits _{ij} {{\varvec{M}_{ij}}} \Vert \varvec{P}_1^{(i)} - \varvec{P}_2^{(j)} \Vert ^2 , \end{aligned}$$

where $\varvec{M}^{m_1\times m_2}$ represents the hierarchical mapping relations between phenotypes at adjacent levels. $\varvec{M}_{ij}$ equals 1 if there is a parent-child association between phenotype *i* and phenotype *j*, and 0 otherwise. The hierarchical mapping constraint minimizes the distances between the phenotypes with parent-child mapping relations in the phenotype ontology *M*. By combining the two components, the loss function is formulated as follows:

4 $$\begin{aligned} & \underset{\varvec{G},{\varvec{P}_1},{\varvec{P}_2}}{\textrm{min}} \left\| \varvec{A}_1 - \varvec{G{P}}_1 \right\| _F^2 + \alpha \left\| {\varvec{A}_2} - \varvec{GP}_2 \right\| _F^2 + \beta \sum \limits _{ij} {{\varvec{M}_{ij}}} \Vert \left(\varvec{P}_1^{(i)}\right) - \varvec{P}_2^{(j)} \Vert ^2 \nonumber \\ & \mathrm {s.t. }\quad \varvec{G}\ge 0,\ \varvec{P}_1\ge 0,\ \varvec{P}_2\ge 0 \end{aligned}$$

where $\alpha>0$ and $\beta>0$ are two hyper-parameters to balance the three components.

### CMNMF algorithm

The loss function in Eq. (4) is not convex on $\varvec{G}$, $\varvec{P}_1$, and $\varvec{P}_2$ jointly, but it is convex on each variable with other variables fixed. Therefore, an alternative iterative schema is adopted, which minimizes the loss function with respect to one variable while fixing the other variables. In the following subsections, the steps of iteratively updating $\varvec{G}$, $\varvec{P}_1$ and $\varvec{P}_2$ are presented separately, and the complete CMNMF algorithm is outlined in Algorithm 1.

#### Updating $\varvec{G}$ with fixed $\varvec{P}_1$ and $\varvec{P}_2$

While variables $\varvec{P}_1$ and $\varvec{P}_2$ are fixed, the partial derivative of Eq. (4) with respect to $\varvec{G}$ is:

$$\begin{aligned} \frac{\partial {L}}{\partial {\varvec{G}}}=-2\left(\varvec{A}_1{\varvec{P}_1^T} - \varvec{G}{\varvec{P}_1}{\varvec{P}_1^T}\right)-2\alpha \left(\varvec{A}_2{\varvec{P}_2^T} - \varvec{G}{\varvec{P}_2}{\varvec{P}_2^T}\right) \end{aligned}$$

and $\varvec{G}$ can be iteratively updated as:

$$\begin{aligned} \varvec{G}_{ij}\leftarrow \varvec{G}_{ij} \frac{\left(\varvec{A}_1\varvec{P}_1^T+\alpha \varvec{A}_2\varvec{P}_2^T\right)_{ij}}{\left(\varvec{GP}_1\varvec{P}_1^T+\alpha \varvec{GP}_2\varvec{P}_2^T\right)_{ij}} \end{aligned}$$

#### Updating $\varvec{P}_1$ with fixed $\varvec{G}$ and $\varvec{P}_2$

While $\varvec{G}$ and $\varvec{P}_2$ are fixed, the partial derivative of Eq. (4) with respect to $\varvec{P}_1$ is:

$$\begin{aligned} \frac{\partial {L\left(\varvec{P}_1\right)}}{\partial {\varvec{P}_1}}= -2\left(\varvec{G}^T\varvec{A}_1-{\varvec{G}^T\varvec{GP}_1}\right)+2\beta \left(\varvec{P}_1\varvec{D}_1- \varvec{P}_2\varvec{M}^T\right) \end{aligned}$$

and $\varvec{P}_1$ can be iteratively updated as:

$$\begin{aligned} (\varvec{P}_1)_{ij}\leftarrow \left(\varvec{P}_1\right)_{ij} \frac{\left(\varvec{G}^T\varvec{A}_1+\beta \varvec{P}_2\varvec{M}^T\right)_{ij}}{\left(\varvec{G}^T\varvec{GP}_1+\beta \varvec{P}_1\varvec{D}_1\right)_{ij}} \end{aligned}$$

#### Updating $\varvec{P}_2$ with fixed $\varvec{G}$ and $\varvec{P}_1$

While $\varvec{G}$ and $\varvec{P}_1$ are fixed, the partial derivative of Eq. (4) with respect to $\varvec{P}_2$ is similar:

$$\begin{aligned} \frac{\partial {L\left(\varvec{P}_2\right)}}{\partial {\varvec{P}_2}}= -2\alpha \left(\varvec{G}^T\varvec{A}_2-{\varvec{G}^T\varvec{GP}_2}\right)+2\beta \left(\varvec{P}_2\varvec{D}_2 - \varvec{P}_1\varvec{M}\right) \end{aligned}$$

and $\varvec{P}_2$ can be iteratively updated as:

$$\begin{aligned} (\varvec{P}_2)_{ij}\leftarrow (\varvec{P}_2)_{ij} \frac{\left(\alpha \varvec{G}^T\varvec{A}_2+\beta \varvec{P}_1\varvec{M}\right)_{ij}}{\left(\alpha \varvec{G}^T\varvec{GP}_2+\beta \varvec{P}_2\varvec{D}_2\right)_{ij}} \end{aligned}$$

#### Normalizing $\varvec{G}$, $\varvec{P}_1$ and $\varvec{P}_2$

For the minimization problem $\min _{\varvec{G},\varvec{P}} \Arrowvert \varvec{A}-\varvec{G}\varvec{P} \Arrowvert _{F}^{2}$, if $\varvec{G}=\varvec{G_0}$ and $\varvec{P}=\varvec{P_0}$ satisfy the minimization condition, then for any invertible matrix $\varvec{X}$, $\varvec{G}=\varvec{G_0}\varvec{X}$ and $\varvec{P}=\varvec{X}^{-1}\varvec{P_0}$ also satisfy the minimization condition. To eliminate the variance of results, the matrix $\varvec{G}$ can be normalized as $\varvec{G}_{ij}\leftarrow \frac{\varvec{G}_{ij}}{\sqrt{\sum _{j}\varvec{G}_{ij}^2}}$, and the matrices $\varvec{P}_1$ and $\varvec{P}_2$ can be normalized as $(\varvec{P}_1)_{ij}\leftarrow \frac{(\varvec{P}_1)_{ij}}{\sqrt{\sum _{j}(\varvec{P}_1)_{ij}^2}}$ and $(\varvec{P}_2)_{ij}\leftarrow \frac{(\varvec{P}_2)_{ij}}{\sqrt{\sum _{j}(\varvec{P}_2)_{ij}^2}}$.

#### Optimization algorithm for CMNMF

The overall optimization algorithm for the loss function of CMNMF is presented as follows:

**Figure:**
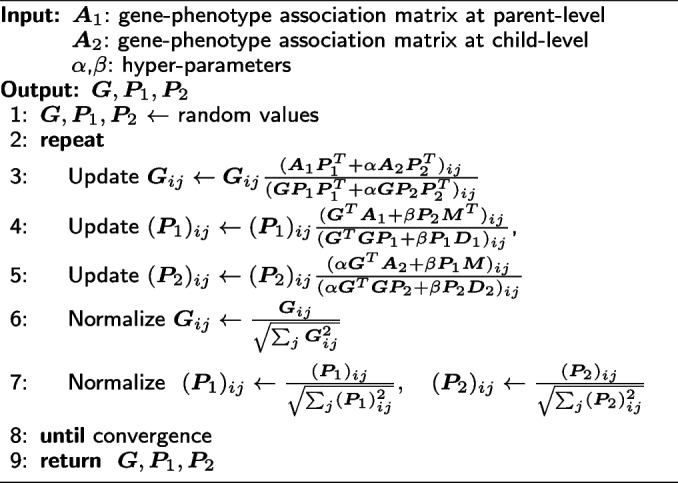
**Algorithm 1** CMNMF

### Parameter tuning

The old versions of PPI network (Feb. 2016) and KEGG pathways (Feb. 2016) were used as validation set to select the best parameters for each method. The models with the best parameters were executed on the new versions of PPI network (Nov. 2020) and KEGG pathways (Nov. 2020) to test their performance.

**Fig. 4:**
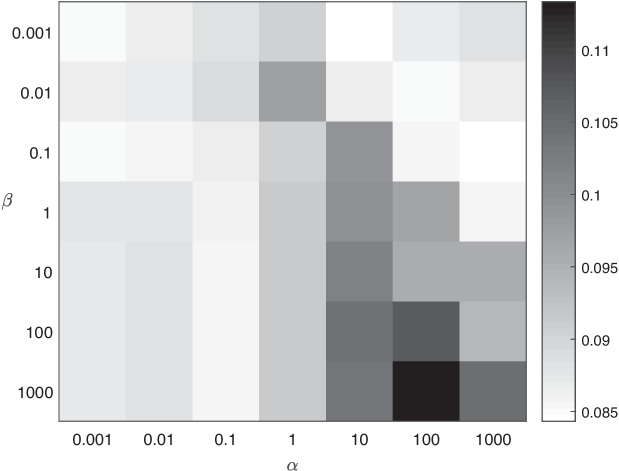
$F_1$ score under different $\alpha$ and $\beta$ combination

Experiments was performed on two biological datasets of mouse species and human species. In the process of parameter tuning, each method was conducted on the validation set repetitively by 10 times and the average evaluation indices were applied for parameter tuning. As the processes of parameter tuning for mouse dataset and human dataset are similar, the details on mouse KEGG pathways are illustrated in Fig. 4.

The hyper-parameters $\alpha$ and $\beta$ were tuned in cross-validation with $F_1$ measure. $\alpha$ balances the contributions of two terms of matrix factorization on different levels of gene-phenotype association matrices. While $\alpha$ turns 0, CMNMF becomes NMF. $\beta$ controls the effects of hierarchical constraint on phenotype ontologies. While $\beta$ is set to 0, CMNMF becomes ColNMF (Collective NMF) [3]. The performance of CMNMF with different combinations of $\alpha$ and $\beta$ on mouse dataset is shown in Fig. 4. The ranges of parameters $\alpha$ and $\beta$ are both set to {0.001, 0.01, 0.1, 1, 10, 100, 1000}, and the darker colors indicates the larger $F_1$ scores under the corresponding combinations of $\alpha$ and $\beta$. In the experiment, CMNMF yielded the best performance while $\alpha =100$ and $\beta = 1000$.

### Evaluation indices

To evaluate the performance of gene clustering, several external indices, including $F_1$ measure, Jaccard Index, Rand index, Precision and Recall are chosen. These metrics were calculated to show the consistency between the learned gene clusters and the known KEGG pathways or the gene pairs in PPI networks. The higher the value is, the more consistent the learned gene clusters are with the known gene sets in KEGG pathways.

### Evaluation with KEGG pathways and PPI networks

In the experiments, the known KEGG pathways and PPI networks are both used to evaluate the performance of clustering. The details are presented as follows:

For any pair of genes, if they are located in common KEGG pathway, they should be clustered into common cluster and vice versa. There are $C_{n}^{2}$ pairs of genes for all *n* genes, according to the correctness of clustering results of these gene pairs, the performance can be evaluated. Similarly, for any pair of genes, if they are associated in true PPI networks, they should be clustered into the same cluster. According to the connectivity of $C_{n}^{2}$ pairs of genes in true PPI network and predicted PPI network, the performance can be evaluated.

## Results

In this section, CMNMF is firstly compared to NMF on a small mouse gene-phenotype association matrix of MGI. Then CMNMF is compared with seven baseline methods by evaluating the consistency between identified functional gene modules and known KEGG pathways or gene pairs in PPI networks.

### CMNMF on a small dataset of mouse gene-phenotype associations

**Fig. 5:**
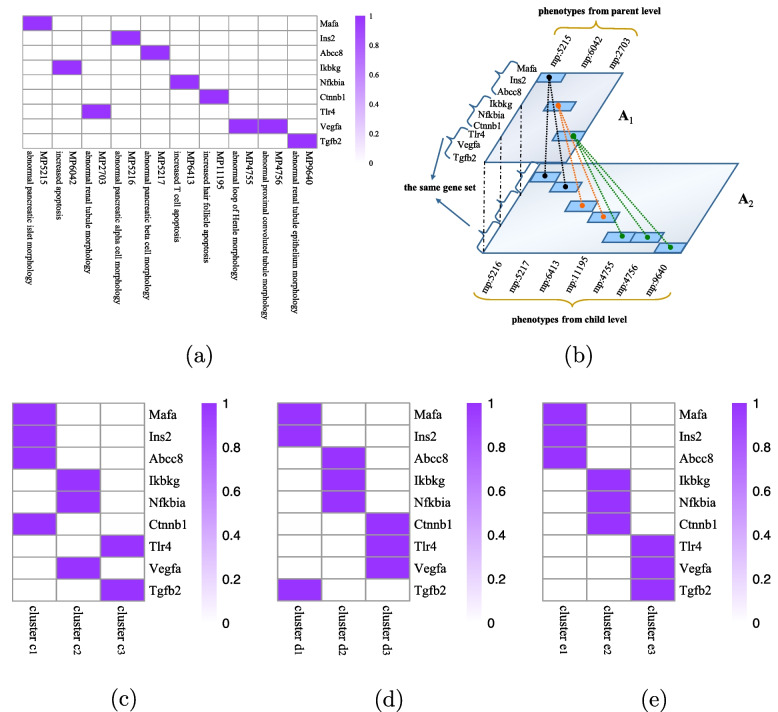
Performance of NMF, CMNMF($\beta$=0) and CMNMF($\beta$=1) on a small gene-phenotype association matrix. **a** Original gene-phenotype association matrix A. **b** Matrix $\varvec{A}$ is divided into $\varvec{A}_1$ and $\varvec{A}_2$ according to the levels of the phenotype ontology. The hierarchical mappings among phenotypes are represented by dot lines. **c** Clustering result of NMF. **d** Clustering result of CMNMF($\beta$=0). **e** Clustering results of CMNMF($\beta$=1)

To illustrate the effect of the consistency constraint (the first two terms in Eq. (4)) and the hierarchical mapping constraint (the last term in Eq. (4)), the performances of NMF, CMNMF($\beta$=0) and CMNMF($\beta$=1) on a small gene-phenotype association matrix from MGI are demonstrated in Fig. 5a. The gene sets in the experiment were selected from three KEGG pathways. In detail, Mafa, Ins2, Abcc8 are from pathway MMU4930 (Type-II diabetes mellitus). Ikbkg, Nfkbia, Ctnnb1 are from MMU5215 (Prostate cancer). Tlr4, Vegfa, Tgfb2 are from MMU5205 (Proteoglycans in cancer). The hierarchical relationships among the phenotypes associated with selected genes are shown in Fig. 5b. A good algorithm should assign the genes from one KEGG pathway into the same cluster.

Figure 5c, d and e indicate the clustering results of NMF, CMNMF($\beta$=0) and CMNMF($\beta$=1), respectively. Compare to Fig. 3c, a significant improvement can be observed in Fig. 5d and e by considering the hierarchical structure of phenotype ontology. By introducing hierarchical constraint among two levels of phenotypes, CMNMF($\beta$=1) assigns gene Ctnnb1 to cluster $e_3$ (Fig. 5e) compare to CMNMF($\beta$=0) (Fig. 5d), and the clustering result of CMNMF($\beta =1$) agrees with gene members in KEGG pathways.

### Comparison with baseline methods by mining functional gene modules

In this section, the gene clusters identified by CMNMF are evaluated with KEGG pathways and PPI network. Seven clustering methods, including agglomerative hierarchical clustering (AHC) [22], agglomerative hierarchical clustering with pairwise constraints [23] (Constrained AHC), K-means, pairwise constrained K-means [24] (Constrained K-means), NMF [25], HMF (Hierarchical Matrix Factorization) [26] and ColNMF (Collective NMF) [3], were compared in the experiment. Since AHC and K-means are unsupervised clustering methods with no parameters, additional pairwise constrained AHC [23] and pairwise constrained K-means [24] were evaluated for a relatively fair comparison with other methods. The old versions of KEGG pathways (Feb. 2016) and PPI network (Feb. 2016) were used as validation sets. For CMNMF, HMF and ColNMF, the gene-phenotype association matrix A were divided into two matrices A1 and A2 according to the levels of phenotype ontologies. For AHC, Constrained AHC, K-means, Constrained K-means, and NMF, the entire gene-phenotype association matrix were applied. For NMF, HMF, ColNMF and CMNMF, the gene clustering results were row-normalized by z-score and $G_{ij}$ was set as 0 if it was less than 3. Five evaluation indices of all methods are reported in Tables 2, 3, 4, 5 and 6.

**Table 2:** Evaluation results on mouse KEGG pathways

	$F_1$ measure	Precision	Recall	Jaccard index	Rand index
AHC	0.0871	0.0624	0.1443	0.0455	0.7654
Constrained AHC	0.0925	0.0554	**0.2803**	0.0485	0.5738
K-means	0.0517	0.0760	0.0392	0.0265	**0.8886**
Constrained K-means	0.0554	0.0750	0.0439	0.0285	0.8839
NMF	0.1037	**0.1084**	0.0993	0.0547	0.8668
HMF	0.0818	0.1080	0.0658	0.0426	0.8855
ColNMF	0.0883	0.1074	0.0750	0.0462	0.8799
CMNMF	**0.1181**	0.0898	0.1726	**0.0628**	0.8002

**Table 3:** Evaluation results on mouse PPI

	$F_1$ measure	Precision	Recall	Jaccard index	Rand index
AHC	0.0037	0.0019	0.1420	0.0019	0.8321
Constrained AHC	0.0080	0.0040	**0.6491**	0.0040	0.6421
K-means	0.0041	0.0022	0.0426	0.0021	0.9544
Constrained K-means	0.0107	0.0056	0.1197	0.0054	0.9509
NMF	0.0126	0.0065	0.1988	0.0063	0.9305
HMF	0.0137	0.0072	0.1298	0.0069	**0.9584**
ColNMF	0.0120	0.0062	0.1420	0.0060	0.9478
CMNMF	**0.0138**	**0.0072**	0.1521	**0.0070**	0.9518

**Table 4:** Evaluation results on human KEGG pathways

	$F_1$ measure	Precision	Recall	Jaccard index	Rand index
AHC	0.0808	0.0998	0.0679	0.0421	**0.9044**
Constrained AHC	0.0952	0.0570	**0.2892**	0.0500	0.6599
K-means	0.0787	0.0829	0.0748	0.0409	0.8915
Constrained K-means	0.0889	0.0813	0.0980	0.0465	0.8756
NMF	0.0966	0.0891	0.1056	0.0508	0.8778
HMF	0.0863	**0.1034**	0.0741	0.0451	0.9029
ColNMF	0.0850	0.0869	0.0831	0.0444	0.8893
CMNMF	**0.1046**	0.0727	0.1886	**0.0552**	0.8023

**Table 5:** Evaluation results on human PPI

	$F_1$ measure	Precision	Recall	Jaccard index	Rand index
AHC	0.0096	0.0055	0.0410	0.0048	0.9709
Constrained AHC	0.0089	0.0045	**0.4430**	0.0045	0.6637
K-means	0.0095	0.0051	0.0743	0.0048	0.9464
Constrained K-means	0.0117	0.0068	0.0422	0.0059	**0.9754**
NMF	0.0141	0.0074	0.1457	0.0071	0.9300
HMF	0.0142	0.0077	0.0927	0.0072	0.9557
ColNMF	0.0156	0.0083	0.1303	0.0079	0.9432
CMNMF	**0.0166**	**0.0089**	0.1203	**0.0084**	0.9510

#### Verification by known KEGG pathways and protein-protein interactions

New versions of KEGG pathways (Nov. 2020) and PPI network (Nov. 2020) were applied to verify the performance of gene clustering. Tables 2 and 3 show the evaluation results on mouse data with KEGG pathways and PPI network, respectively. The evaluation results on human data are reported in Tables 4 and 5. The best results across all the methods are bold. Compared with the baseline methods, CMNMF outperforms other methods on $F_1$ measure and Jaccard Index in all cases on both mouse and human data. It demonstrates the advantage of imposing the consistency constraint on two levels of gene clusters and the hierarchical constraint on the clusters of hierarchically related phenotypes. In particular, ColNMF introduces additional knowledge from the consistency constraint, thus defeating conventional NMF. Moreover, CMNMF adopts the hierarchical constraint of phenotypes to reinforce the phenotype clusters to describe the hierarchical relation in phenotype ontology, so CMNMF gets better performance compared with ColNMF (CMNMF with $\beta =0$). However, AHC works better than other methods in index “Recall”. The clustering results of AHC were analyzed and a few large-scale gene clusters (with more than four hundred genes) were found, which would result in an increase in index “Recall” and a decrease in index “Precision”. The paradox in AHC is caused by its centroid criterion, which tends to find the clusters that leads to minimal increase in total inter-cluster Euclidean distances when it merges the clusters. Therefore, the compact clustering results of AHC will benefit the “Recall” index. It is also noticed that CMNMF does not achieve the best performance on “Rand Index”. As known, “Rand Index” takes true negative gene pairs into consideration. However, the experimental results were evaluated on true positive gene pairs, i.e., the known common genes in one cluster. True negative gene pairs are dominant in the original data (accounting for 95%−99% of all gene pairs), which would lead to a biased comparison between different methods. Overall, CMNMF outperforms all compared clustering methods and the improvement is significant.

#### Verification by the latest protein-protein interactions

CMNMF was also tested on the latest protein-protein interactions added from Feb. 2016 to Nov. 2020 in BIOGRID. The parameters $\alpha$ and $\beta$ were tuned by the old version of PPI mentioned in the previous section with $F_1$ measure. The results are reported in Tables 6 and 7 for mouse and human respectively. Compared with baseline methods, CMNMF also outperforms them on $F_1$ measure, Jaccard Index and Precision, which proves its splendid effectiveness to predict unknown Protein-Protein Interactions.

**Table 6:** Evaluation results on the latest mouse PPI

	$F_1$ measure	Precision	Recall	Jaccard index	Rand index
AHC	0.0019	0.0009	0.1023	0.0009	0.8677
Constrained AHC	0.0025	0.0012	**0.7112**	0.0012	0.3076
K-means	0.0019	0.0009	0.2500	0.0009	0.6796
Constrained K-means	0.0019	0.0009	0.0708	0.0009	0.9105
NMF	0.0043	0.0022	0.0969	0.0021	0.9456
HMF	0.0039	0.0021	0.0427	0.0020	**0.9738**
ColNMF	0.0044	0.0022	0.0927	0.0022	0.9489
CMNMF	**0.0045**	**0.0023**	0.1235	**0.0023**	0.9341

**Table 7:** Evaluation results on the latest human PPI

	$F_1$ measure	Precision	Recall	Jaccard index	Rand index
AHC	0.0065	0.0035	0.0394	0.0033	0.9613
Constrained AHC	0.0065	0.0033	**0.3755**	0.0033	0.6339
K-means	0.0058	0.0030	0.0737	0.0029	0.9184
Constrained K-means	0.0074	0.0041	0.0357	0.0037	**0.9691**
NMF	0.0106	0.0055	0.1336	0.0053	0.9199
HMF	0.0111	0.0057	0.1689	0.0056	0.9033
ColNMF	0.0106	0.0055	0.1351	0.0054	0.9193
CMNMF	**0.0115**	**0.0061**	0.1118	**0.0058**	0.9384

## Discussion

### GO enrichment analysis

In this section, Gene Ontology enrichment analysis was performed to evaluate the biological significance of discovered gene modules.

The functional roles of the identified gene clusters were studied with enrichment analysis against Gene Ontology (GO) using DAVID [16]. The enriched GO terms involving selected gene clusters are reported in Table 8, the *p*-value and FDR adjusted *p*-value are also presented in the table. It is clear that gene clusters found by CMNMF are biologically functionally relevant.

**Table 8:** Enrichment analysis on gene clusters mined by CMNMF with Gene Ontology

No	Gene cluster	Most related GO terms	*P*-value	FDR
294	ND5, ND2, COX1, COX2, CYTB,ND1, ND4,COX3,*etc*	mitochondrial electron transport, NADH to ubiquinone;	1.30E-12	6.20E-11
		mitochondrial respiratory chain complex I assembly;	1.20E-09	2.90E-08
		ATP synthesis coupled electron transport;	6.40E-06	1.00E-04
216	CALM3,RYR2,SCN10A, AKAP9,CAV3,KCNJ, SCN5A,CALM1, *etc*	regulation of heart rate by cardiac conduction;	1.40E-26	3.30E-24
		regulation of ventricular cardiac muscle cell membrane repolarization;	3.30E-25	4.00E-23
		ventricular cardiac muscle cell action potential;	9.10E-20	7.30E-18
149	ITGA2,ITGA2B,ITGB3, MYH9,GP6,CD109, CD36,ANKRD49, *etc*	blood coagulation;	1.90E-19	6.60E-17
		platelet aggregation;	4.30E-09	7.20E-07
		platelet activation;	1.90E-08	2.10E-06
53	NDUFA1,NUDFA2,NDUFS1, NUDFS4,NDUFV1,NDUFV2, SLC25A26,ATP5F1E, *etc*	mitochondrial respiratory chain complex I assembly;	1.70E-61	1.40E-59
		mitochondrial electron transport, NADH to ubiquinone;	9.90E-45	4.00E-43
		reactive oxygen species metabolic process;	1.50E-06	4.10E-05
12	PTPN11,RAF1,SOS1, SOS2,LZTR1,CBL, RASA2,RAF1, *etc*	Ras protein signal transductionn;	1.30E-07	1.90E-05
		small GTPase mediated signal transduction;	4.70E-07	3.30E-05
		neurotrophin TRK receptor signaling pathway;	5.60E-05	2.60E-03

The first column is the index of gene clusters identified by CMNMF. For each gene cluster, a part of genes are presented in the second column. Three most related GO terms, corresponding *p*-values and false discovery rate (FDR) are reported in the following columns

By matching the corresponding gene clusters, the genes in No.294 cluster are found to be associated with Parkinson disease. Heteroplasmic mutations in a specific region of ND5 largely segregated Parkinson’s disease from controls and might be of major pathogenic importance in idiopathic Parkinson’s disease [27]. Ksel found a heteroplasmic mtDNAG5460A missense mutation in the ND2 subunit gene of NADH dehydrogenase was three times more frequent in Parkinson cases (4/21) compared to controls (5/77). In Ksel’s study, he provided a hint that the ND25460 mutation, in combination with other factors, could play a role in disease pathogenesis in a subset of Parkinson patients [28]. Teismann used acetylsalicylic acid, a COX-1/COX-2 inhibitor, to study the possible role of the isoenzymes of cyclooxygenase COX-1 and COX-2 in the MPTP mouse model of Parkinson’s disease [29]. Tanaka analyzed amino acid variations in the cytochromeb (CYTB) molecule of 64 Japanese centenarians and found out the most striking feature of centenarian CYTB was the rareness of amino acid variations in contrast to the variety of amino acid replacements in patients with Parkinson’s disease [30].

By matching the corresponding gene cluster, the genes in No.216 cluster are found associated with Alzheimer disease. In patients with late onset Alzheimer’s disease (AD), the calcium buffering capacity of lymphoblasts is reduced. Calmodulin is a calcium binding protein codified by three genes, one of them (CALM3) maps to chromosome 19, nearby a gene, apoE, associated with late onset AD [31]. Yao found that Neuronal hyperactivity is an early primary dysfunction in Alzheimer’s disease (AD) in humans and animal models, but pharmacologically limiting RyR2 open time with the R-carvedilol enantiomer prevents and rescues neuronal hyperactivity, memory impairment, and neuron loss even in late stages of AD [32]. Soo showed that SCN10A (Sodium channel protein type 10 subunit alpha), a target of bupivacaine, was linked with MAPT (tau) and PSEN1 (presenilin1), which were associated with the pathological hallmarks of AD, via SCN1A (sodium channel protein type 1 subunit alpha), a target of topiramate [33]. Logue identified by whole exome sequencing a small number of AA AD cases and subsequent genotyping in a large AA sample of AD cases and controls association of AD risk with a pair of rare missense variants in AKAP9 [34].

### Analysis on identified gene clusters of CMNMF and ColNMF

To verify the practical effectiveness of CMNMF to cluster genes, each gene cluster identified by CMNMF and ColNMF is analyzed via DAVID Tools, which can reveal the most relevant GO terms correlated with the corresponding group of genes. The results of all clusters are thoroughly analyzed and some typical clusters are presented as follows:

**Table 9:** The Most Relevant GO Terms of Typical Clusters Identified by CMNMF and ColNMF

	CMNMF			ColNMF		
	GO term	*P*-value	FDR	GO term	*P*-value	FDR
No. 48	ossification	1.4e-5	1.9e-3	glycosaminoglycan	3.1e-4	5.9e-2
				catabolic process		
	glycosaminoglycan	1.4e-5	1.9e-3	porphyrin-containing	8.1e-3	4.3e-1
	catabolic process			compound biosynthetic process		
	endochondral	1.4e-5	1.9e-3	peptidyl-lysine	8.1e-3	4.3e-1
	ossification			monomethylation		
	keratan sulfate	2.2e-4	2.1e-2	protoporphyrinogen IX	9.1e-3	4.3e-1
	catabolic process			biosynthetic process		
	lysosome	2.2e-3	1.5e-1	middle ear	2.2e-2	6.7e-1
	organization			morphogenesis		
No. 57	cell surface receptor	8.9e-6	5.2e-3	glycosaminoglycan	1.5e-2	8.1e-1
	signaling pathway			catabolic process		
	immune response	1.7e-5	5.2e-3	glycosaminoglycan	1.7e-2	8.1e-1
				metabolic process		
	positive regulation of	2.1e-5	5.2e-3	glycosaminoglycan	2.5e-2	8.1e-1
	T cell proliferation			biosynthetic process		
	negative regulation of	2.9e-5	5.3e-3	retinoid metabolic	3.6e-2	8.1e-1
	neuron apoptotic process			process		
	apoptotic signaling pathway	4.2e-5	6.1e-3	lung development	4.4e-2	8.1e-1
No. 61	extracellular	1.7e-3	1.4e-1	brain	1.2e-2	4.9e-1
	matrix-cell signaling			development		
	retinal blood	1.7e-3	1.4e-1	positive regulation	1.3e-2	4.9e-1
	vessel morphogenesis			GTPase activity		
	negative regulation	5.8e-3	3.2e-1	preassembly of	1.3e-2	4.9e-1
	of cell-substrate			GPI anchor in		
	adhesion			ER membrane		
No. 102	isotype switching	2.0e-9	7.3e-7	transition between	4.8e-4	1.9e-2
				slow and fast fiber		
	somatic hypermutation	8.0e-7	1.5e-4	-	-	-
	of immunoglobulin					
	genes					
	mismatch repair	9.2e-6	1.1e-3	-	-	-
	cell cycle arrest	6.0e-4	5.5e-2	-	-	-
	peptidyl-tyrosine phosphorylation	7.5e-4	5.6e-2	-	-	-

Table 9 presents the most relevant GO Terms correlated with 4 typical gene clusters (No. 48, 57, 61 and 102) identified by CMNMF and ColNMF, where the columns “*P*-value” and “FDR” indicate the relevance between GO terms and the group of genes, and lower values mean higher relevance. The gene clusters identified by CMNMF possess significantly higher cohesiveness than ColNMF since they have tighter associations with GO terms.

### Paired T-tests between CMNMF and each baseline under F1 scores and Jaccard Indices

To demonstrate the real performance enhancement of CMNMF over baselines, paired *t*-tests are implemented between CMNMF and each baseline in 10-fold cross-validation under F1 scores and jaccard indices. Comparisons on clustering 4 datasets by paired t-test are reported in Additional file 1: Table S1-S4. Clearly, CMNMF performs significantly better than other baselines.

According to the results in 4 tables, the F1 and Jaccard indices of CMNMF are significantly better than all baselines in human pathway and mouse pathway datasets, while they are significantly better than most baselines in human PPI and mouse PPI datasets, which proves the real performance enhancement of CMNMF over baselines.

## Conclusions

Our results indicate that the gene clusters identified by CMNMF are biologically relevant with the additional information from phenotype ontology hierarchical structure. The evaluation experiments on KEGG pathways and PPI networks of mouse and human data show the advantage of CMNMF over other baseline methods. The experiment on the latest PPI dataset show the ability of identifying new protein-protein interactions. Furthermore, the gene modules identified by CMNMF are enriched in biological relevant functions.

## Supplementary Information

Supplementary Material 1.

## Data Availability

All data used in this paper is downloaded from open access datasets. A MATLAB software package is available through Github at https://github.com/nkuiip/CMNMF, containing all the source codes used to run CMNMF.

## Abbreviations

NMF: Nonnegative matrix factorization
CMNMF: Consistent multi-view nonnegative matrix factorization
AHC: Agglomerative hierarchical clustering
ColNMF: Collective nonnegative matrix factorization
GO: Gene Ontology
MGI: Mouse Genome Informatics
PPI: Protein-protein interactions
HPO: Human Phenotype Ontology
KEGG: Kyoto Encyclopedia of Genes and Genomes
DAVID: The Database for Annotation, Visualization and Integrated Discovery
