# Supplementary material for: Mining functional gene modules by multi-view NMF of phenome-genome association
Source: BMC Genomics. 2025 Jan 9;23(Suppl 6):868. doi: 10.1186/s12864-024-11120-5 (PMC11720361; doi:10.1186/s12864-024-11120-5)
Supplement: Supplementary file 1 — Supplementary Material 1. [file 12864_2024_11120_MOESM1_ESM.pdf]

Additional File 1: Table S1-S4

September 28, 2024

Table S1: A Pair-Wise Comparison by Paired T-Tests on Clustering Results in 10-Fold Cross-Validation Based on F1 Scores and Jaccard Indices on Human KEGG Pathway Dataset.

Human KEGG Pathway				
	F1 Score		Jaccard Index	
	Hypothesis	*p*-value	Hypothesis	*p*-value
CMNMF>AHC	1	6.70e-10	1	6.19e-10
CMNMF>Constrained AHC	1	2.21e-04	1	2.04e-04
CMNMF>K-means	1	2.52e-06	1	2.19e-06
CMNMF>Constrained K-means	1	4.47e-10	1	3.77e-10
CMNMF>NMF	1	2.10e-05	1	2.28e-05
CMNMF>HMF	1	2.98e-05	1	3.07e-05
CMNMF>ColNMF	1	1.92e-05	1	2.09e-05

Table S2: A Pair-Wise Comparison by Paired T-Tests on Clustering Results in 10-Fold Cross-Validation Based on F1 Scores and Jaccard Indices on Mouse Pathway Dataset.

Mouse Pathway				
	F1 Score		Jaccard Index	
	Hypothesis	*p*-value	Hypothesis	*p*-value
CMNMF>AHC	1	1.84e-10	1	2.65e-10
CMNMF>Constrained AHC	1	4.56e-07	1	4.68e-07
CMNMF>K-means	1	3.77e-08	1	4.33e-08
CMNMF>Constrained K-means	1	1.87e-11	1	3.12e-11
CMNMF>NMF	1	3.82e-08	1	4.56e-08
CMNMF>HMF	1	7.12e-12	1	1.47e-11
CMNMF>ColNMF	1	2.58e-09	1	3.32e-09

Table S3: A Pair-Wise Comparison by Paired T-Tests on Clustering Results in 10-Fold Cross-Validation Based on F1 Scores and Jaccard Indices on Human PPI Dataset.

Human PPI				
	F1 Score		Jaccard Index	
	Hypothesis	p-value	Hypothesis	p-value
CMNMF>AHC	1	6.00e-03	1	5.98e-03
CMNMF>Constrained AHC	1	6.86e-11	1	1.07e-10
CMNMF>K-means	1	1.60e-09	1	1.78e-09
CMNMF>Constrained K-means	0	8.26e-01	1	8.10e-01
CMNMF>NMF	1	2.67e-07	1	1.68e-07
CMNMF>HMF	0	1.00e-00	1	1.00e-00
CMNMF>ColNMF	1	6.93e-07	1	4.73e-07

Table S4: A Pair-Wise Comparison by Paired T-Tests on Clustering Results in 10-Fold Cross-Validation Based on F1 Scores and Jaccard Indices on Mouse PPI Dataset.

Mouse PPI				
	F1 Score		Jaccard Index	
	Hypothesis	p-value	Hypothesis	p-value
CMNMF>AHC	1	1.08e-11	1	1.17e-11
CMNMF>Constrained AHC	1	2.66e-11	1	1.53e-10
CMNMF>K-means	1	1.22e-11	1	5.15e-11
CMNMF>Constrained K-means	1	1.01e-10	1	2.56e-10
CMNMF>NMF	0	1.00e-00	1	1.00e-00
CMNMF>HMF	0	8.66e-01	1	9.21e-01
CMNMF>ColNMF	0	1.00e-00	1	1.00e-00
